# Long-Term Visual Field Progression in X-Linked Retinitis Pigmentosa Patients

**DOI:** 10.3390/diagnostics14242797

**Published:** 2024-12-12

**Authors:** Alvilda Hemmingsen Steensberg, Sermed Al-Hamdani, Michael Stormly Hansen, Oliver Niels Klefter, Mette Bertelsen, Steffen Hamann

**Affiliations:** 1 Department of Ophthalmology, Copenhagen University Hospital—Rigshospitalet, 2600 Glostrup, Denmarksteffen.ellitsgaard.hamann@regionh.dk (S.H.); 2 Department of Clinical Medicine, University of Copenhagen, 1172 Copenhagen, Denmark; 3 Department of Clinical Genetics, Copenhagen University Hospital—Rigshospitalet, 2100 Copenhagen, Denmark; 4 Neuro-Ophthalmology Department, Rothschild Foundation Hospital, 75019 Paris, France

**Keywords:** retinitis pigmentosa, X-linked, visual field, perimetry, Goldmann

## Abstract

We present an image that illustrates long-term visual field progression in patients with X-linked retinitis pigmentosa (XLRP) due to the retinitis pigmentosa GTPase regulator (RPGR) and retinitis pigmentosa 2 protein (RP2) gene variants. Longitudinal data from 84 genetically confirmed XLRP patients were collected from the Danish Retinitis Pigmentosa Registry, spanning the years 1948 to 2014. A visual field summation image revealed the characteristic pattern of retinal degeneration and visual field preservation in XLRP.

**Figure 1:**
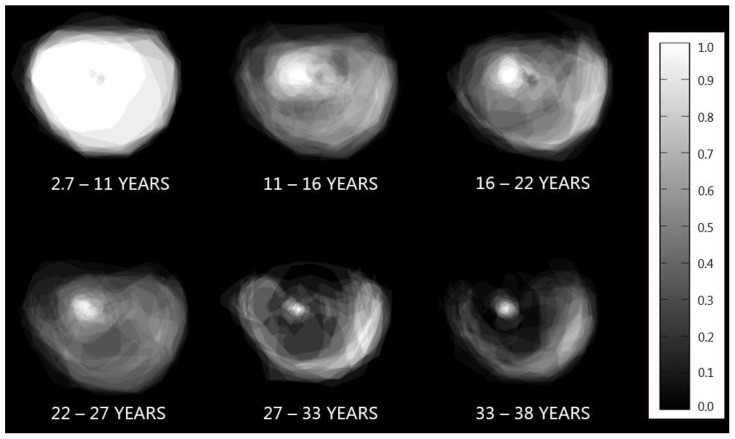
A visual greyscale representation of long-term visual field progression in the right eye of 84 patients with X-linked retinitis pigmentosa (XLRP) caused by the retinitis pigmentosa GTPase regulator (RPGR) and retinitis pigmentosa 2 (RP2) variants. These are the two most common genes involved in XLRP, with the RPGR and RP2 variants being responsible for 74% and 15% of cases, respectively [1,2]. Data from patients with genetically confirmed XLRP and at least one follow-up test of Goldmann kinetic perimetry were retrospectively collected in a longitudinal manner from the Danish Retinitis Pigmentosa Registry, covering 1948 to 2014. The study included 84 XLRP patients (RPGR: 57, RP2: 27), with a mean follow-up time of 14.6 years, resulting in 1185 patient-years of observation. The kinetic visual fields were obtained using a V4e light stimulator on a standard Goldmann perimetry (Haag-Streit AG, Koeniz, Switzerland) background light (31.5 apostilbs). The Goldmann planimetric visual fields were digitally mapped for the right eye in each test session. Across the specified time periods, these visual fields were overlaid to generate a composite visualization of the visual field progression over the years. To the right, a greyscale reference bar illustrates the varying function of the overlapping visual fields. White (1.0) depicts regions where all visual fields exhibit function, black (0.0) depicts regions where no visual fields have function, and shades of grey reflect intermediate levels of functional visual fields. The loss of visual field with the preservation of a temporal crescent until the advanced stages, a common characteristic in RP progression, is evident, reflecting the retinal degeneration.
